# Pre-Existing Interstitial Lung Abnormalities in Patients with Head and Neck Squamous Cell Carcinoma and Their Follow Up after Therapy

**DOI:** 10.3390/diagnostics13182908

**Published:** 2023-09-11

**Authors:** Danica Vuković, Danijela Budimir Mršić, Kristian Jerković, Benjamin Benzon, Tade Tadić

**Affiliations:** 1 Clinical Department of Diagnostic and Interventional Radiology, University Hospital Split, Šoltanska 2, 21000 Split, Croatia; dvukovic@kbsplit.hr (D.V.); dbudmrs@kbsplit.hr (D.B.M.); ttadic@kbsplit.hr (T.T.); 2 School of Medicine, University of Split, Šoltanska 2, 21000 Split, Croatia; benjamin.benzon@mefst.hr; 3 University Department of Health Studies, University of Split, Ruđera Boškovića 35, 21000 Split, Croatia

**Keywords:** ILA, head and neck squamous cell cancer, lung MSCT, Fleischner Society guidelines, adjuvant radiotherapy

## Abstract

Interstitial lung abnormalities (ILAs) are incidentally found nondependent parenchymal abnormalities affecting more than 5% of any lung zone and are potentially related to interstitial lung disease and worsening post-treatment outcomes in malignancies and infectious diseases. The aim of this study was to determine the prevalence and type of ILA changes in patients with head and neck squamous cell carcinoma (HNSCC) and their change in the follow-up period. This retrospective single-center study included 113 patients with newly diagnosed HNSCC who underwent lung MSCT prior to treatment. ILAs were reported in 13.3% of patients on pretreatment MSCT. Patients with ILAs were significantly older (median 75 vs. 67 years). ILAs were most prevalent in lower zones (73.3%) (*p* = 0.0045). The most reported ILA subtype was subpleural non-fibrotic (60%) (*p* = 0.0354). Reticulations were the most frequently described pattern (93.3%) (*p* < 0.0001). Progression of ILAs was reported in almost 30% of patients after receiving therapy. Patients with pre-existing ILAs were more likely to develop radiation-induced lung fibrosis after adjuvant radiotherapy (*p* = 0.0464). In conclusion, ILA’s incidence, distribution and presentation were similar to previous research conducted in other special cohorts. Our research suggests a possible association of more frequent radiation pneumonitis with ILA changes in patients with HNSCC, which should be further investigated.

## 1. Introduction

Squamous cell carcinomas of the head and neck (HNSCCs) develop from the mucosal epithelium of the oral cavity, pharynx and larynx and represent the most common malignancies in this region. Rarely, cancers can also arise in the salivary glands, sinuses, muscles or nerves of the head and neck regions, but these types are much less common compared to squamous cell carcinoma [1,2]. Sometimes, cancerous squamous cells can be found in the lymph nodes of the upper neck without evidence of a primary cancer, possibly because the primary tumor is too small. In this case, the cancer is called a metastatic squamous cell carcinoma with unknown (occult) primary location [3,4]. HNSCCs have generally been associated with exposure to tobacco-derived carcinogens, excessive alcohol consumption or both [1,2]. Although some tumors of the pharynx have also been linked to prior infection with certain oncogenic strains of human papillomavirus (HPV), primarily HPV-16, the majority of the HNSCCs are strongly associated with smoking and are collectively referred to as HPV-negative HNSCC [1,2,5].

Besides HNSCCs, tobacco smoking could also be one of the causes of interstitial lung abnormalities (ILA) [6,7]. ILAs include nondependent parenchymal abnormalities affecting more than 5% of any lung zone. The findings consist of ground-glass or reticular abnormalities, lung distortion, traction bronchiectasis or bronchiolectasis, honeycombing and non-emphysematous cysts [6,7,8,9]. ILA can be subcategorized according to the distribution and the presence of fibrosis according to Fleischner Society guidelines, that is, non-subpleural (NS), subpleural fibrotic (SF) and subpleural non-fibrotic (SNF) distributed [6,9]. Fibrosis is characterized by the presence of architectural distortion with traction bronchiectasis or bronchiolectasis and/or honeycombing [6,7,10]. Risk factors for the presence of ILA, other than cigarette smoking, include increasing age, other inhalational exposures (e.g., vapors, gases, dust, fumes, traffic-related air pollution such as nitrogen oxides or carbon), some biomarkers and genetic factors [6,10,11,12,13,14]. Regarding genetic factors, a piece of evidence has been found that associates HLA-DRB1*14 allele with increased risk for developing ILA, especially those of subpleural location [15]. The systematic evaluation of large cohorts has shown a prevalence of ILA in 2–9% of cigarette smokers and 2–7% of non-smokers [7,16]. ILA does not imply the presence of respiratory symptoms or functional impairment. Those findings are potentially compatible with interstitial lung disease (ILD). ILA can possibly progress to ILD in some cases. ILD is composed of lung diseases with overlapping clinical, radiological, physiological and pathological features [6,9]. Differential diagnosis of ILA includes pulmonary edema, lymphangitis carcinomatosis, atelectasis and dependent lesions, which can all be resolved according to the medical history of the patient but also with the dynamic of the disease. Furthermore, if the patient has rheumatoid arthritis, systemic sclerosis or familial ILD, interstitial lung changes are not classified as ILA because there is a known link between mentioned entities and interstitial disease (ILD) [6,7,8,9,10].

Several studies conducted on lung cancer patients have suggested an association between the presence of pretreatment ILAs and cancer-associated mortality, found on both patients with early-stage cancer treated with surgical resection, as well as the patients with stage 4 disease treated with systemic therapy [17,18,19]. In addition, pre-existing ILA is associated with an increased risk of radiation pneumonitis grade III or higher in patients treated with radiotherapy [18,20]. Further, immune checkpoint inhibitors (ICI) are first-line therapies for patients with advanced malignancies, including HNSCCs. Approximately 5% of patients treated with ICI show drug-related pneumonitis, and it has been shown that the incidence of pneumonitis was significantly higher in patients with pre-existing ILA than in those without ILA [21,22,23,24]. Moreover, patients with ILA changes are described as having a greater risk of developing acute respiratory distress caused by positive pressure ventilation while undergoing surgery [7]. To our knowledge, compared to ILAs in lung cancer patients, the incidence and type of ILA in HNSCC patients have not been broadly investigated. We think this possibly might help clinicians with the careful monitoring of HNSCC patients with ILA prior to or during cancer treatment by looking for respiratory symptoms (cough, decreased total lung capacity, shortness of breath, reduced exercise capacity), blood changes (oxygen, carbon dioxide) and radiological changes in lung parenchyma, such as organizing pneumonia or drug and radiation pneumonitis, to prevent worse outcomes or serious complications in patients with ILA changes. Therefore, the primary objective of the current study was to investigate the prevalence and type of incidental changes in lung parenchyma categorized as ILA in patients with HNSCC and their possible alteration following different cancer treatment protocols.

## 2. Materials and Methods

### 2.1. Participants and Study Design

This retrospective observational study was conducted at a tertiary radiology department of an academic medical center between May and June of 2023. We retrospectively analyzed pretreatment lung multi-slice computed tomography (MSCT) for ILA changes in patients with newly diagnosed HNSCC. Date of diagnosis of HNSCC was between September 2019 and September 2022. Lung MSCT was performed shortly before or after the time of diagnosis. The patient’s medical history was retrieved from the Hospital Information System (HIS). The inclusion criteria were as follows: (1) all patients who were 18 years old or older at the time of diagnosis, patients of all sexes and all races; (2) all stages of HNSCC. The exclusion criteria were as follows: (1) patients with incomplete medical documentation; (2) patients with diagnosis of HNSCC who did not undergo lung MSCT at the time of diagnosis due to localized disease; (3) patients who underwent lung MSCT after receiving systemic therapy (immunotherapy, chemotherapy) or known localized therapy of lung region (lung surgery or radiotherapy) for any reason; (4) patients with any other malignancy synchronous with the diagnosis of HNSCC with lung involvement or lung metastasis. Furthermore, we analyzed ILA changes in lung parenchyma on follow-up lung MSCT performed after treatment of HNSCC available at the time of conducted study (May and June 2023).

### 2.2. Analysis of Imaging Data

Lung scanning was performed using the 128-slice MSCT Siemens Somatom Definition AS, Germany. All patients were requested to hold their breath at maximal inspiration in the supine position. MSCT parameters used were tube current of 113 to 200 mAs, tube voltage of 120 kVp, automatically adjusted depending on a patient’s physical characteristics. Non-contrast scans and scans in venous phase (approximately 60 s delay) were performed with lung parenchyma reconstructions from non-contrast scans. Lung MSCT imaging data were separately reviewed by two experienced radiologists (T.T. and K.J.) with more than twenty (T.T.) and ten years (K.J.) of expertise in the field of thoracic radiology, in a random order, blinded to the original scan report using the Fleischner Society guidelines. Each reader analyzed and recorded the presence or absence of the following interstitial abnormalities: ground-glass opacity (GGO) or reticular abnormalities, lung distortion, traction bronchiectasis or bronchiolectasis, honeycombing and non-emphysematous cysts. Subsequently, patients with reported abnormalities were subdivided into groups by the distribution and the type of change: non-subpleural, subpleural fibrotic and subpleural non-fibrotic [7,8]. Interclass correlation coefficient (ICC) between two independent observers (radiologists) was 1, which means a total agreement for all findings described, except in case of three patients with GGO (ICC 0.51; 95% CI −0.26–0.82). This disagreement was resolved by consensus.

### 2.3. Statistical Analysis

Categorical variables are presented as percentage; continuous ones are presented as median and interquartile range (IQR). Fisher’s exact test was used to examine differences in categorical variables and Mann–Whitney U test and T-test for numerical ones. The statistical significance threshold was set to *p* < 0.05. Analysis was conducted in Past 4.11 software [25].

### 2.4. Institutional Rewiev Board Statement

Institutional Review Board approval was obtained, and this study was conducted under all ethical principles of the Seventh Revision of the Helsinki Declaration from 2013. Due to the retrospective nature of this study, informed consent was waived.

## 3. Results

**Figure 1:**
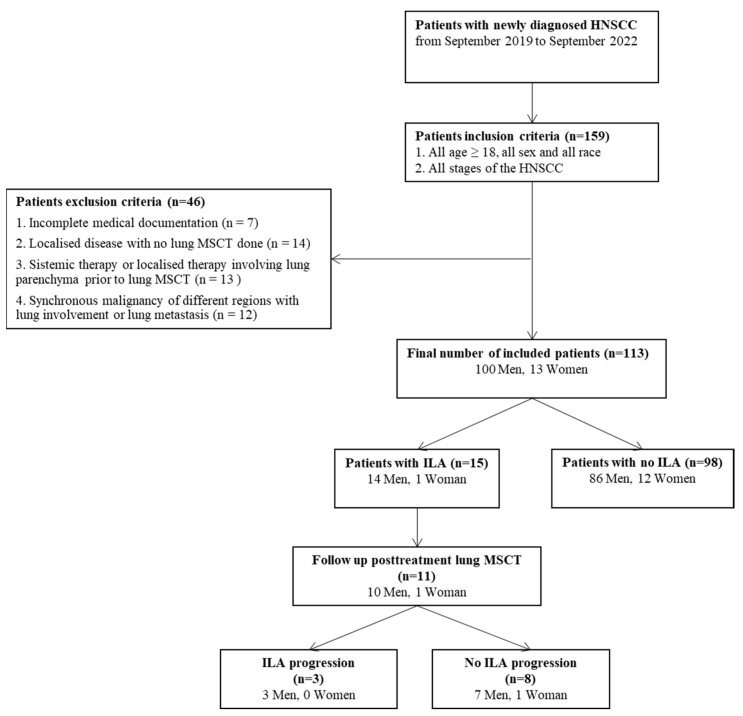
Flow chart of the study population included.

Initially, 159 patients with newly diagnosed HNSCC were identified through electronic search of the HIS. Twelve patients with synchronous cancer of different regions, 14 patients who did not undergo lung MSCT (patients with localized disease), 7 patients with incomplete medical documentation and 13 patients who received therapy prior to lung MSCT (chemotherapy, radiotherapy, lung surgery or immunotherapy) for any medical condition were excluded from further analysis due to the mentioned reasons (Figure 1). A final number of 113 patients was enrolled in this study, of which 100 (88.5%) were men and 13 (11.5%) were women, with a median age of 75 years (IQR 65–75) in the ILA group and a median age of 67 years (IQR 61–71.25) in the non-ILA group (*p* = 0.03) (Figure 1). ILA changes were reported in 15 (13.3%) patients.

From the total number of included patients in this study, 101 patients (89.3%) are former or current cigarette smokers, and 13 patients (11.5%) were alcohol consumers with no statistically significant difference in smoking and alcohol status between the ILA and non-ILA groups. The number of cigarettes consumed per day was available for 49 patients, and all were classified as heavy smokers (more than 20 cigarettes per day), with no statistically significant difference between the groups (*p* = 0.3878). The most frequent comorbidity in the HNSCC cohort was arterial hypertension (*n* = 26, 23%), followed by chronic obstructive lung disease (COPD) (*n* = 10, 8.8%) and diabetes type 2 (*n* = 10, 8.8%). Previously diagnosed cancer, with no involvement of the lungs or therapy which could cause lung parenchymal changes, was identified in 12 patients, as a localized (*n* = 10, 8.8%) or metastatic (*n* = 2, 1.8%) disease (Table 1).

A significant difference in the observed demographic and clinical variables between the ILA and non-ILA groups was found in cases of patients’ ages (*p* = 0.0300) and hypertension (*p* = 0.0418): ILA patients were older and had a higher incidence of hypertension. On the contrary, no differences in other variables were found, as follows: sex *p* = 0.5283, alcohol consumption *p* = 0.8115, tobacco smoking status *p* = 0.1979, earlier localized malignancies *p* = 0.6198, earlier metastatic disease *p* = 0.5767, diabetes mellitus type 2 *p* = 0.6186 and COPD *p* = 0.6198 (Table 1).

**Figure 2:**
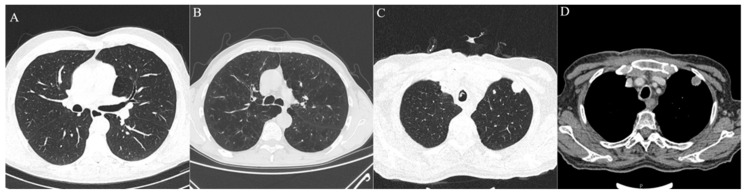
Axial MSCT scans of pretreatment lung parenchyma demonstrating other radiological findings found in HNSCC patients. (**A**) Healthy lung parenchyma in lung window reconstruction. (**B**) Advanced centrilobular emphysema affecting all lung lobes seen as hyperlucent lung parenchyma, typical finding for heavy smokers in lung window reconstruction. (**C**,**D**) Same patient with lung metastasis which presents as soft tissue density, well circumscribed, round lesion with significant contrast enhancement and central necrosis in lung window reconstruction (**C**) and soft tissue window with intravenous contrast (**D**).

Analysis of lung MSCT found that the most frequently reported radiological findings on MSCT scans were centrilobular (*n* = 34, 30%) and paraseptal (*n* = 36, 31.9%) emphysema, centrilobular nodules (*n* = 21, 18.6%), pleural plaques (*n* = 2, 1.8%) and calcification of coronary arteries (*n* = 91, 81.4%). There was no significant statistical difference in reported radiological findings between the ILA and non-ILA groups, except pleural plaques, which were reported only in the ILA group (*p* = 0.0165). Lung metastasis was observed in nine patients (7.96%) with no statistical difference between the groups (*p* = 0.341) (Table 2, Figure 2).

**Figure 3:**
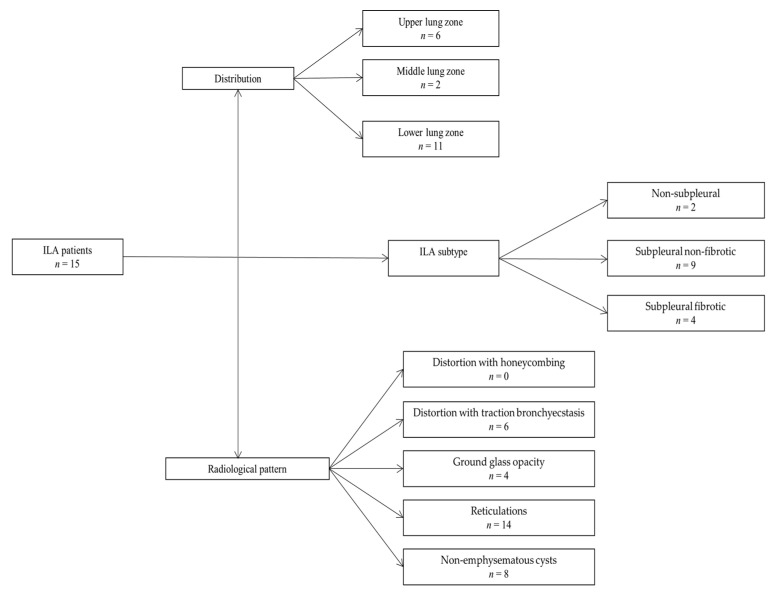
Chart demonstrating patients with HNSCC with reported ILA changes classified according to ILA distribution, ILA subtype and radiological pattern.

**Figure 4:**
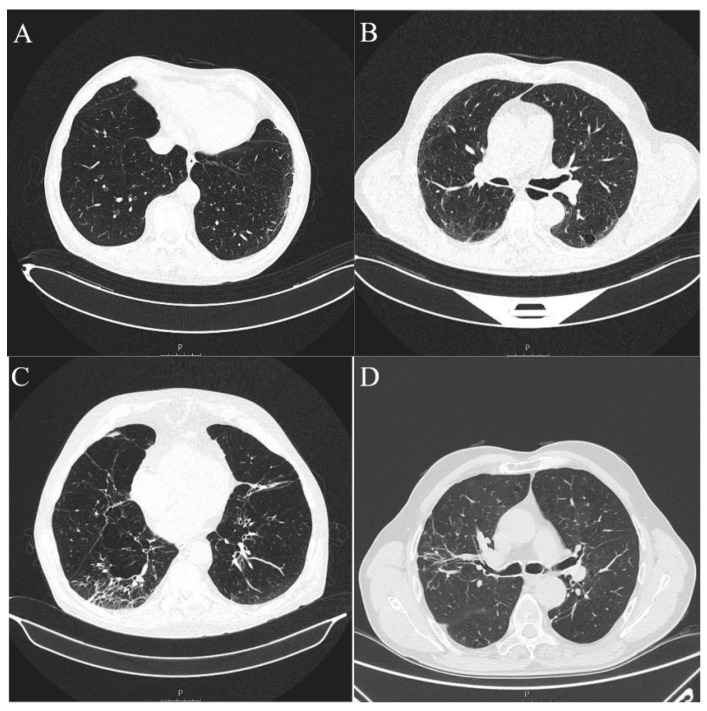
Axial MSCT scans of pretreatment lung parenchyma demonstrating typical ILA findings in HNSCC patients (**A**). Unilateral subpleural non-fibrotic type characterized by subpleural reticulations surrounded by areas of GGO (**B**). Bilateral subpleural non-fibrotic changes manifesting as subpleural reticulations surrounded by areas of GGO and sporadic non-emphysematous cysts (**C**). Unilateral subpleural fibrotic changes in the right lung, manifesting as traction bronchiectasis and bronchiolectasis with parenchymal distortion and zones of GGO. Contralateral lung shows mild traction bronchiectasis and subpleural reticulations (**D**). Unilateral non-subpleural, traction bronchiectasis with architectural distortion surrounded by small zones of GGO. Two small intraparenchymal calcifications in close proximity were not classified as ILA.

ILA changes were most prevalent in the lower lung zones (*n* = 11, 73.3%), followed by upper zones (*n* = 6, 40.00%) and least reported in the middle lung zones (*n* = 2, 13.3%), which was significantly different (*p* = 0.0045). The most commonly reported ILA subtype was subpleural non-fibrotic (*n* = 9, 60%), followed by subpleural fibrotic (*n* = 4, 26.67%) and non-subpleural (*n* = 2, 13.33%) (*p* = 0.0354) (Table 3, Figure 3 and Figure 4). Reticulations were the most frequently described pattern in almost every patient with ILA changes (*n* = 14, 93.3%) (*p* < 0.0001). Other ILA patterns were non-emphysematous cysts (*n* = 8, 53.33%), distortion with traction bronchiectasis (*n* = 6, 40.00%) and ground glass opacity in four (26.67%) patients. Distortion with honeycombing was not reported in any of the patients (Table 4, Figure 3 and Figure 4).

The correlation between the observed clinical parameters and the affected lung zone with ILA changes was established only for arterial hypertension, that is, there is a 75% probability that a patient with ILA changes in the lower lung zone will have arterial hypertension (*p* = 0.0338) (Table 5).

**Figure 5:**
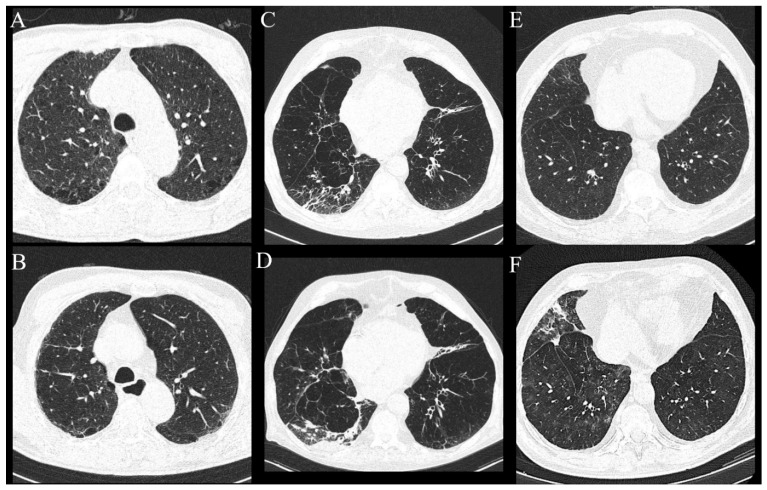
Axial MSCT scans of lung parenchyma demonstrating progression of existing and newly developed findings in HNSCC patients after cancer treatment. (**A**,**B**) Patient had subpleural non-fibrotic ILA subcategory in the lower lung zones, but middle lung zones were not involved (as shown on the picture), and only paraseptal emphysema was visible. In the follow-up period, the patient demonstrated newly developed subpleural reticulations with zones of GGO, which was characterized as probable UIP pattern. (**C**,**D**) Same patient with progression of subpleural fibrotic ILAs manifested as fibrous consolidations in the right lower lobe and newly developed subpleural reticulations which persisted on subsequent scans. (**E**,**F**) Patient with progression of subpleural non-fibrotic ILA now seen as fibrotic consolidations, reticulations and areas of GGO. Patient had centrilobular nodules diffusely arranged throughout lung parenchyma, which became more prominent.

The follow-up lung MSCT scans were available for 65 out of 113 patients, of which 11 were patients with ILAs (11 of a total number of 15 with ILAs), within a median of 24 months (Q1-Q3 13–32 months) from the baseline lung MSCT performed. Progression of existing ILA changes was described in three patients (27.27%). The first patient, after having surgical resection and adjuvant chemoradiotherapy, developed new non-emphysematous cysts and more prominent subpleural reticulations in all lung zones with areas of GGO and was characterized as a possible usual interstitial pneumonia (UIP) pattern. The second and third patients, after having surgical resection followed by adjuvant radiotherapy, developed fibrous consolidations with GGO areas and newly developed reticulations (Figure 5). All patients had subpleural ILAs in the lower lung zones.

**Figure 6:**
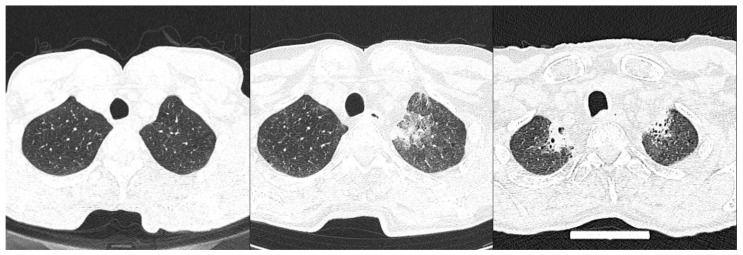
Axial MSCT scans of lung parenchyma in patients with HNSCC and reported ILA changes in lower lung zones after adjuvant radiotherapy, demonstrating (left to right): upper lung zone prior to treatment showing no abnormality; following acute radiation injury of lung shown as GGO; thickened intralobular and interlobular septa; and finally fibrous consolidations with architectural distortion in the last picture.

Out of 65 patients with an available follow-up lung MSCT, 21 patients received adjuvant radiotherapy of regional lymph nodes (60Gy in 30 fractions) after initial surgical resection. There was statistically significant difference in radiation-induced fibrosis in the upper lung zones after adjuvant radiotherapy in the ILA compared to non-ILA group (Table 6, Figure 6).

## 4. Discussion

Our findings demonstrate that ILA changes are a relatively common finding in the newly diagnosed HNSCC cohort, as they were present in 13.3% of patients. Tobacco smoking is a well-known risk factor for developing malignancies of various regions including HNSCC and also one of the risk factors for ILA changes, which could partially explain this incidence. Some studies even found association with the amount of smoking (packs per year) with airway remodeling in patients with fibrotic ILA but also lower diffusing capacity for carbon monoxide in heavy tobacco smokers [26]. Other risk factors for ILA include increasing age, other inhalational exposures and genetic factors. As for age, in our study, patients with ILA changes were statistically significantly older than the non-ILA group, which is completely in congruency with the current literature [7,10]. One study showed that the risk of interstitial lung disease development increased with age, and the risk was 6.9 times higher in those aged over 70 than in their forties [27]. We did not find any statistically significant difference in ILA incidence in women in comparison with men, but some studies have found male sex to be a risk factor for developing ILA [6].

Pre-existing ILA changes were described in other malignancies, in a similar percentage, such as in lung cancer observed during screening trials or pretreatment MSCT (7.8% up to 15%) but also locally advanced esophageal cancer (7%) [19,28,29]. ILA was also described in a population older than 60 years, in whom it ranged from 9% in tobacco smokers to 7% in non-smokers [9,28,29,30]. Because aging and tobacco smoking are important risk factors for ILA, the prevalence of ILA in our study could be explained by an overall high number of older patients who were cigarette smokers included in this study. Moreover, a large number of patients who had localized disease did not perform lung MSCT in their diagnostic work-up and were therefore excluded from this study, which might also contribute to the prevalence of ILA.

There was no significant statistical difference in the majority of clinical and other radiological findings between the ILA and the non-ILA groups, except arterial hypertension which was more commonly described in the ILA group, and it is in congruence with finding of increased age in ILA patients. The chances of developing arterial hypertension increase with age. Furthermore, pleural plaques were reported only in the ILA group. Other studies have found no significant difference in the presence of pleural plaques between the ILA and the non-ILA groups [29]. Regarding our study, one patient had a medical history of exposure to asbestos materials but did not have the diagnosis of asbestosis, and the other one had tuberculosis many years ago, so these could be the reasons for our findings.

Lower lung zones were the most commonly reported zones to have ILA changes, which is similar to other studies [6,9,29], and ILAs were the least reported in the middle lung zones. The subpleural non-fibrotic subtype was the most frequently described, with reticulations being the most frequently described pattern. Radiologists agreed with the description of all ILA changes (ICC = 1), except for three cases of GGO (ICC 0.51; 95% CI −0.26–0.82). The debate was whether these were real GGO changes or dependent hypoventilation changes (supine position of patient or inadequate inspiration). This disagreement was resolved by consensus. ILA changes are described as lung parenchymal damage due to inflammation and fibrosis [24], and the lower lung zones are the most common sites of infectious and aspiration pneumonia but also hydrostatic edema due to hemodynamic heart dysfunction, which could explain lower zone predominance [31].

Progression of ILA has been described in some populations with ILA, especially in those with fibrotic ILA changes. However, one study also showed that one half of subpleural non-fibrotic ILAs progressed radiologically over 4 years, which led to the conclusion that the presence of reticulations in non-fibrotic ILA is also an important risk factor for progression [32]. We found a prevalent subpleural non-fibrotic type of ILA, and 72.73% of the described ILA changes did not progress over time, considering a relatively short follow-up period of 24 months, which is in accordance with the literature [7,33]. Both patients in whom the progression of ILAs was described had a subpleural location of ILA changes in the lower lung zones and received adjuvant radiotherapy or concurrent chemoradiotherapy. Radiotherapy and chemotherapy are known to cause radiopathological findings on the lung parenchyma, but their impact on pre-existing ILA and the dynamic of their progression in time is yet to be investigated, although there is some evidence in favor [19,34]. One of our patients with progression of ILA was later described as a probable UIP pattern. Therapy-induced transition from ILA to ILD requires further research. There are some from large clinical lung cancer screening cohorts that show a link between ILA detected in baseline MSCT and all-cause mortality, with a significant number of patients eventually receiving ILD diagnosis [35,36]. Some studies, with larger cohorts, showed that ILA had progressed in up to 80% of patients over a period of 8 years, with fibrotic ILA being the dominant subtype [33]. The progression of ILA changes has even been linked, in some cases, to MUC5B (mucin 5B, oligomeric mucus/gel-forming), a genetic marker linked with increasing risk for pulmonary fibrosis [10,14].

Newly developed lung changes in the upper lung zones, described as radiation-induced lung fibrosis, were a more frequently described radiological finding after the use of adjuvant radiotherapy of regional lymph nodes in the ILA group. Radiation pneumonitis is described as an acute manifestation of radiation-induced lung disease with consequential radiation-induced pulmonary fibrosis if not resolved [19]. Although the images within the interval of 3 months after receiving radiotherapy were not available for the analysis and assessment of acute radiation-induced changes, more frequent incidence of fibrosis in the upper zones might be an indicator that the occurrence or extent of post-radiation changes is related to ILA changes in patients with HNSCC. There is some evidence that ILA, in fact, may contribute to post-therapy changes in the lung parenchyma. Numerous studies have revealed ILA changes to be an independent risk factor for worsening post-treatment outcomes in lung and esophageal malignancies.

Research on the non-small lung cell carcinoma (NSCLC) cohort revealed that individuals with interstitial lung changes had a higher risk of developing immune-related pneumonia following immunotherapy [37]. Additionally, in patients with NSCLC receiving immune checkpoint inhibitor monotherapy, the presence of non-fibrotic ILAs was noted as a significant risk factor for early onset immune checkpoint inhibitor-induced interstitial lung disease (ICI-ILD) [38]. In stage III of NSCLC patients who have received definitive concurrent chemoradiotherapy but still have unresectable disease, pretreatment fibrotic ILA has been shown to be strongly related with symptomatic radiation pneumonitis and poor survival [39].

ILA changes are linked to worse prognosis not only in malignancies but also in infectious diseases. A study conducted by Colombi et al. showed that patients affected by COVID-19 pneumonia with pre-existing fibrotic ILAs had worse prognosis, with a 2.7 times increased risk of intensive care unit (ICU) admission or death [40]. This increasing incidence of adverse events could be explained by inadequate or changed/fibrotic lung parenchyma being considered as a locus minoris resistentiae, as it inhibits immune responses to external stressors [41].

The main strength of this study is that, to our knowledge, this was the first research of interstitial lung abnormalities on baseline lung MSCT in the head and neck cancer cohort as well as their alteration after therapy and possible impact on late radiation-induced lung injury. Because HNSCC therapy includes surgery, radiotherapy, chemotherapy and immunotherapy [42], we think that detailed evaluation of pretreatment lung MSCT and evaluation of ILA changes should be considered because of earlier described worsening outcomes in patients after treatment.

The most important limitations of our study are the relatively small sample size and retrospective study design. All MSCT scans were scanned in a supine position. The follow-up period for detection of progression of changes was relatively short; most of the previous studies performed control MSCT scans within a minimum of two years. Our research suggests a possible influence of ILA changes on the development of radiation-induced fibrosis visible by imaging. However, the information on the size of the radiation field was not accessible, and we did not take into account radiation intervals, as well as possible interruptions in radiation therapy, and the number of total patients included in the follow-up was quite small. The above-mentioned details reduce the scientific strength of this evidence. Larger cohort prospective studies are required to monitor the impact of radiotherapy on ILA changes in lung parenchyma in HNSCC patients together with functional lung tests and blood work. We suggest that future research link radiological findings (the type and involvement of the lung parenchyma with ILA changes) with functional lung tests.

## 5. Conclusions

To conclude, we found incidental lung changes on MSCT scans, classified as ILA, in newly diagnosed HNSCC patients undergoing lung MSCT prior to initiation of treatment. ILA’s incidence, distribution and presentation on MSCT scans were similar to the previous research conducted in special cohorts of patients, which advocates for their possible progression in the longer follow-up period; thus, they might have an impact on the patient’s clinical outcome. Higher incidence of radiation-induced fibrosis in upper lung zones in ILA patients suggests a possible association of more frequently developed radiation pneumonitis in patients with ILA changes with HNSCC. While ILA is a potentially important prognostic factor, in a population of head and neck carcinoma they were not broadly investigated, and their prognostic significance is yet to be investigated.

## Figures and Tables

**Table 1:** Clinical findings in ILA and non-ILA groups in patients with HNSCC.

	ILA (*n* = 15)	Non-ILA (*n* = 98)	*p* Value
	*n* (%)	*n* (%)	
Age (years, IQR)	75 (65–75)	67 (61–71.25)	0.0300 *
Sex (Male)	14 (93.33)	86 (87.76)	0.5283 ^#^
Alcohol consumption	2 (13.33)	11 (11.22)	0.8115 ^#^
Tobacco smoking statusNumber of cigarettes per day, *n* = 49 (IQR)	12 (80.00) 30 (20–50)	89 (90.82) 40 (20–40)	0.1979 ^#^ 0.3878 ^†^
Earlier localized malignancy	2 (13.33)	8 (8.16)	0.6198 ^#^
Earlier metastatic malignancy	0 (0)	2 (2.04)	0.5767 ^#^
Diabetes mellitus	2 (13.33)	8 (81.63)	0.6198 ^#^
Arterial hypertension	7 (46.67)	19 (19.39)	0.0418 ^#^
COPD	2 (13.33)	8 (8.16)	0.6198 ^#^
Region of cancer			
Oral cavity	3 (20.00)	34 (34.69)	0.3781 ^#^
Pharynx	5 (33.33)	29 (29.59)	0.7684 ^#^
Larynx	7 (46.67)	33 (33.67)	0.3886 ^#^
Salivary glands	0	1 (1.02)	1 ^#^
Unknown origin	0	3 (30.61)	1 ^#^

* Mann–Whitney U test; ^#^ Fisher’s exact test; ^†^ *t*-test; COPD—chronic obstructive pulmonary disease.

**Table 2:** Radiological findings on lung parenchyma observed in pretreatment lung MSCT scans in ILA and non-ILA groups in patients with HNSCC.

	ILA (*n* = 15)	Non-ILA (*n* = 98)	*p* Value *
	*n* (%)	*n* (%)	
Centrilobular emphysema	7 (46.67)	27 (27.55)	0.1429
Paraseptal emphysema	8 (53.33)	28 (28.57)	0.0744
Centrilobular nodules	5 (33.33)	16 (16.33)	0.1504
Pleural plaques	2 (13.33)	0 (0)	0.0165
Coronary calcifications Lung metastasis	13 (86.67) 2 (13.33)	78 (79.59) 7 (7.14)	0.7315 0.341

* Fisher’s exact test.

**Table 3:** ILA distribution patterns and subtypes in ILA group found on pretreatment lung MSCT in HNSCC patients.

	Lower Zone	Middle Zone	Upper Zone	*p* Value *
	*n* (%)	*n* (%)	*n* (%)	
Present	11 (73.33)	2 (13.33)	6 (40.00)	0.0045
	**Non-subpleural**	**Subpleural fibrotic**	**Subpleural non-fibrotic**	
	*n* (%)	*n* (%)	*n* (%)	
Present	2 (13.33)	4 (26.67)	9 (60.00)	0.0354

* Fisher’s exact test.

**Table 4:** ILA radiological patterns group observed on pretreatment lung MSCT in HNSCC patients.

	Distortion with Honeycombing	Distortion with Traction Bronchiectasis	Ground Glass Opacity	Non-Emphysematous Cysts	Reticulations	*p* Value *
	*n* (%)	*n* (%)	*n* (%)	*n* (%)	*n* (%)	
Present	0 (0.00)	6 (40.00)	4 (26.67)	8 (53.33)	14 (93.33)	<0.0001

* Fisher’s exact test.

**Table 5:** Correlation between lung zone and clinical parameters observed in HNSCC patients with ILA changes.

	Upper Zone	Middle Zone	Lower Zone	*p* Value *
	*n* (%)	*n* (%)		
Arterial hypertension	1 (12.5)	1 (12.5)	6 (75)	0.0338
Diabetes mellitus	1 (33.33)	1 (33.33)	1 (33.33)	1
COPD	0 (0)	0 (0)	2 (100)	0.3182

* Fisher’s exact test.

**Table 6:** Radiation-induced fibrotic lung changes in the upper lung zones in ILA and non-ILA groups at the follow-up lung MSCT after receiving therapy.

	ILA (*n* = 6 of 11 Follow-Up MSCT in the ILA Group)	Non-ILA (*n* = 15 of 54 Follow-Up MSCT in Non-ILA Group)	*p* Value
	*n* (%)	*n* (%)	
Age (years, IQR)	74 (70–74)	70 (63–77)	0.1584 ^#^
Sex (male)	5 (83.33)	12 (80.00)	1 *
Radiation-induced lung fibrosis	5 (83.33)	4 (26.67)	0.0464 *
Region of cancer			
Oral cavity	2 (33.33)	5 (33.33)	1 *
Pharynx	1 (16.67)	2 (13.33)	1 *
Larynx	3 (50.00)	8 (53.33)	1 *

^#^ *t*-test; * Fisher’s exact test.

## Data Availability

The datasets are available upon reasonable request to the corresponding author.
